# Tryptophan as a Central Hub for Host/Microbial Symbiosis

**DOI:** 10.1177/1178646920919755

**Published:** 2020-05-11

**Authors:** Monica Borghi, Matteo Puccetti, Marilena Pariano, Giorgia Renga, Claudia Stincardini, Maurizio Ricci, Stefano Giovagnoli, Claudio Costantini, Luigina Romani

**Affiliations:** 1 Department of Experimental Medicine, University of Perugia, Perugia, Italy; 2 Department of Pharmaceutical Science, University of Perugia, Perugia, Italy

**Keywords:** Tryptophan, indoleamine 2, 3-dioxygenase 1, aryl hydrocarbon receptor, fungal infections, metabolic syndrome, indoles

## Abstract

Amino acid catabolism occurs during inflammation and regulates innate and adaptive immunity. The role of commensal bacteria in amino acid catabolism and the production of metabolites able to regulate the development and function of the innate immune system is increasingly being recognized. Therefore, commensal bacteria are key players in the maintenance of immune homeostasis. However, the intestinal microbiota also contributes to susceptibility and response to infectious diseases. This is self-evident for fungal infections known to occur as a consequence of weakened immune system and broad-spectrum antibiotic use or abuse. Thus, diseases caused by opportunistic fungi can no longer be viewed as dependent only on a weakened host but also on a disrupted microbiota. Based on these premises, the present review focuses on the role of amino acid metabolic pathways in the dialogue between the mammalian host and its microbiota and the potential implications in fungal commensalism and infectivity.

## Introduction

The cross-talk between the mammalian host and pathogens is driven by 2 opposite forces, the balance of which determines the outcome of the interaction.^1^ On one hand, the host puts in place resistance mechanisms directed against the pathogen to prevent the infection and promote the clearance. On the other hand, the host uses tolerance to defend rather than attack, a strategy aimed at reducing the damage induced by the pathogen or by the host itself, in the case of an exaggerated immune response.^2^ Thus, while the formers constitute a true act of force, the latter more likely resembles a trade-off. As a corollary, resistance may confer protection against infection, but not tolerance^3^ because the pathogens continue to persist in a relationship that may take the form of an “apparent commensalism.”^4^ Whatever the mechanism, the interaction with pathogens is associated with energy costs^5^ that come from shared nutrient requirements between the host and the pathogens. An additional player is represented by commensal microorganisms that may compete for the same nutrients or use indigestible food, such as in the production of short-chain fatty acids that play multiple beneficial roles in the host, by providing an energy source to colonocytes and regulating the mucosal immune response.^6^ Therefore, nutrient utilization emerges as a fundamental aspect in the triad interaction among the host, the pathogens, and the commensal microorganisms, a competition that may leave its mark on the outcome of the cross-talk among all the players.

Amino acid metabolic pathways are crucial regulators of immunity from plants ^7^ to mammals.^8^ Indeed, not only amino acids are the building blocks of proteins, including the ones essential for the activation and function of the immune system, but they also directly participate in the antimicrobial response.^9^ In mammals, l-arginine is a semi-essential amino acid involved in protein biosynthesis and as an intermediate in the liver urea cycle during homeostasis. In immune responses, metabolism of l-arginine in activated myeloid cells occurs by 2 principal pathways: nitric oxide synthase (NOS)-mediated production of nitric oxide (NO) or arginase (Arg)-mediated production of l-ornithine. Competition of inducible nitric oxide synthase (iNOS) and Arg for the same substrate and prevalence of one pathway over the other may affect the outcome of microbial infection.^10^ For instance, neutrophils and macrophages deploy oxidative and nitrosative killing mechanisms against *Candida* by means of iNOS expression^11^ and NOS inhibitors may reduce fungal damage in the lung.^12^ *Candida* has evolved a defense strategy to divert arginine utilization away from NO production by inducing its own Arg^10^ or increasing Arg1 activity in macrophages by means of chitin.^13^ While being important for *Candida* resistance and tolerance, the effect of arginine metabolism in the response to *Aspergillus* is disputed. Indeed, NOS and Arg1 expression in macrophages are not involved in conidiocidal activity^14^ and allergic inflammation induced by *Aspergillus*,^15^ respectively. *Aspergillus fumigatus* is endowed with a system for detoxification of host-derived reactive nitrogen intermediates, although it does not seem to contribute to the fungus virulence.^16^

While current knowledge is limited on the impact of l-arginine metabolism in the outcome of fungal infections, a great wealth of information is accumulating on tryptophan (Trp) metabolism and downstream molecules with profound implications in the communication between the host immune system, its microbiome, and pathogens and will be the focus of this review.

## Tryptophan as a Central Hub for Host/Microbial Information Processing

Tryptophan is an essential amino acid for humans and must be obtained from the diet. Besides being involved in protein synthesis, Trp is a versatile precursor and can be metabolized by both host^17^ and microbial^18^ enzymes to generate a variety of molecules involved in different fundamental processes. Two pathways have gained considerable interest for their role at the interface between the host, the microbiome, and pathogens, namely the host kynurenine pathway and the microbial indole pathway, that converge on a central xenobiotic receptor, the aryl hydrocarbon receptor (AhR), a critical regulator at the host/microbe interface. Other pathways clearly influence the interaction between host and microbes, including the Trp-to-serotonin pathway. For instance, commensal bacteria regulate the synthesis of serotonin by the host,^19^ and serotonin may modulate the composition of the gut microbiome.^20^ In addition, serotonin may influence the virulence of the pathogen *Pseudomonas aeruginosa*.^21^ However, the role of this pathway is outside the scope of the present review that is centered on the host and microbial Trp metabolites that converge of AhR.

### The mammalian Trp-to-kynurenine pathway

The kynurenine pathway accounts for ~95% of overall Trp degradation,^17^ and the first and rate-limiting step is mediated by indoleamine 2, 3-dioxygenase (IDO)1, along with IDO2 (a paralogue of IDO1) and the tryptophan 2,3-dioxygenase, TDO2,^17^ resulting in the formation of *N*-formylkynurenine, then hydrolyzed to kynurenine by the activity of an *N*-formylkynurenine formamidase. Although the kynurenine pathway leads to various metabolic intermediates with a role in the host-microbe interaction,^22^ kynurenine and IDO1 have emerged for their critical function in the promotion of tolerance in different inflammatory conditions,^23-26^ and will be the object of this chapter. For instance, the Trp metabolic pathway crucially provides immune homeostasis in fungal infections by taming heightened inflammatory responses and inducing immune and tissue tolerance, an activity to which the host, fungi, and the microbiota cooperatively contributed.^27-29^ On elucidating the relative contribution of the different mammalian dioxygenases in antifungal immunity and tolerance, it was found to be dependent on the combined effects of TDO2, IDO1, and AhR, a ligand-operated transcription factor activated by l-kynurenine. Thus, the enzymatic activity of IDO1 has 2 immediate effects: on one hand, it deprives the local environment of Trp, and, on the other hand, it produces kynurenine, a bioactive metabolite able to activate AhR. AhR, in turn, plays multiple roles. Indeed, besides the long-known function as a xenobiotic receptor, AhR has been implicated in a wide range of physiological activities, including the bidirectional communication with the microbiome for tuning host immunity, tolerance, and metabolism.^30-32^ For instance, AhR regulates interleukin (IL)-22 expression and helps maintaining epithelial barrier function and intraepithelial lymphocytes.^33^ These activities help ensure that commensal bacteria outcompete pathogenic bacteria in the gut microbiota, allowing AhR to mediate host-microbe homeostasis.^33^

The IDO1/AhR pathway could be exploited therapeutically in mice and human preclinical settings by a number of immune modulators and cytokines. We have shown that genetic deficiency of pentraxin 3, a soluble pattern recognition receptor that activates IDO1 through the Toll-like receptor4/TIR-domain-containing adapter-inducing interferon-β-dependent immune pathway,^34^ affected resistance and tolerance to *A fumigatus* and contributed to the risk of invasive aspergillosis in patients undergoing hematopoietic stem cell transplantation.^35^ More recently, the IL-9/Th9 axis was found to provide IDO1-dependent tolerance to fungi in the gut and lung via mast cells.^36-38^ In murine and human long-term granulomatous disease and cystic fibrosis, monogenic disorders highly susceptible to pulmonary infections,^39,40^ and recurrent vulvovaginal candidiasis, characterized by exaggerated inflammation associated with symptomatic infection,^41^ we have shown that (1) IDO1 and tolerance were both defective, (2) the IL-1β/inflammasome system was hyperactivated, and (3) restoring IDO1 activity reinstalled antifungal tolerance while decreasing pathogenic Th17 activation.^42-47^ Therefore, host metabolic pathways, such as the IDO1-dependent Trp catabolic pathway, might be actively pursued as potential druggable targets for antifungal tolerance defenses.

The relationship between IDO and microbes, including fungi, is multifaceted. On one hand, microbes may induce IDO1 to promote downregulation of the immune response and pathogen colonization. On the other hand, IDO1 activation may locally induce a condition of Trp starvation that is detrimental to Trp auxotroph microbes.^29,48^ However, the situation may be more complex. For instance, *Mycobacterium tuberculosis* can synthetize Trp on starvation, thus efficiently counteracting the protective mechanism put in place by the host.^49^ As a matter of fact, IDO is either required^50^ or not^51^ for the immunological control of the infection. Microbes can also indirectly regulate the activation of IDO1 by altering the environmental conditions. For instance, during an inflammatory response, the hypoxic environment that is generated inhibits the activity of IDO1 and prevents its antimicrobial activity.^52^

Finally, IDO1 can cross-talk with the adaptive immune response triggered by the presence of fungi.^29^ Indeed, on one hand, the Th17 pathway and IDO1 negatively regulate each other. Indeed, the former inhibits the Trp metabolism, and the consequent tolerance breakdown promotes opportunistic fungal infections in the presence of long-term inflammation, while activation of IDO1 induces the differentiation of Treg cells that dampen the Th17-mediated inflammation. On the other hand, interferon gamma (IFNγ) is a known activator of IDO1 and can promote tolerance to enable fungal persistence. Therefore, an intricate relationship exists between fungi and the tolerogenic pathway induced by IDO1, that points to Trp metabolites as crucially contributing to the interkingdom dialogue.^29^

### The microbial Trp-to-indole pathway

Immunity at mucosal surfaces is a delicate balance between resistance and tolerance to the microbes. In the healthy mucosa, the endogenous microbiome regulates the immune system by means of several mechanisms.^53^ Lack of Trp of the diet has been shown to impair intestinal immunity and to alter the gut microbial community,^54^ indicating that different factors contribute to mucosal homeostasis and the metabolism of Trp plays an important regulatory role. Consistent with the notion that less than 1% of ingested Trp would be used for serotonin synthesis, metabolic pathways targeting the Trp can lead to a myriad of metabolites, of both host and microbial origins, the largest group of which involves indole and indole derivatives.^55,56^ Production of indoles may be a general property of eubacteria, and coevolution of indole-producing bacteria with animals over the last ∼500 My may explain how indoles coordinate responses to a myriad of stressors in such diverse organisms. Bacterial synthesis of indole compounds occurs via different metabolic pathways mainly involving the activity of (1) tryptophanase generating indole, (2) aromatic amino acid aminotransferase (ArAT) generating indole-3-acetaldehyde and indole-3-aldehyde (3-IAld, also referred to as ICA), and (3) tryptophan deaminase generating indole-3-pyruvic acid. Indoles are very attractive molecules as they have been shown to augment health span across a broad range of evolutionarily diverse species from different phyla^57^ and to control bacterial fitness, including antibiotic resistance.^58^ Microbiota-derived indoles are ligands of AhR,^18^ thus suggesting that the host AhR has evolved to sense and respond to the presence of the microbiota resulting in maintenance of homeostasis.^31,33,59-61^

**Figure 1.**
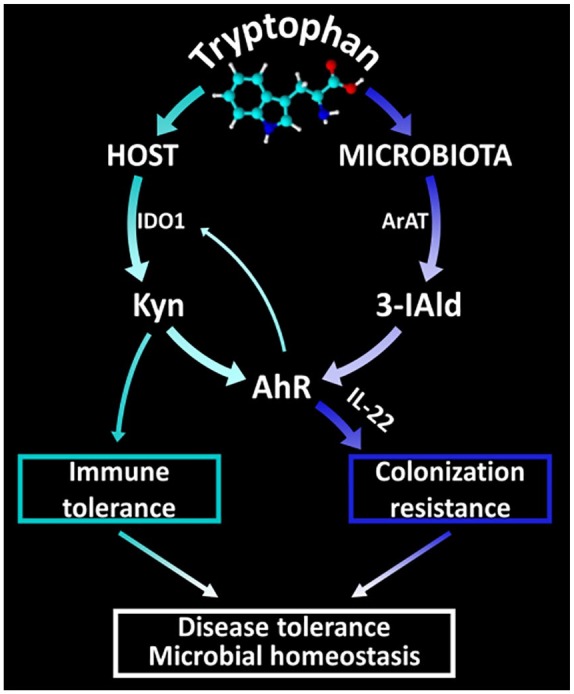
Schematic depiction of the host and microbial pathways of tryptophan utilization. 3-IAld indicates indole-3-aldehyde; AhR, aryl hydrocarbon receptor; IDO1, indoleamine 2, 3-dioxygenase 1; IL-22, interleukin 22. The panel shows that tryptophan utilization via the IDO1 pathway leads to tolerance and activation of AhR by kynurenines. The panel also shows that tryptophan utilization by microbes leads to the generation of indole derivatives, including 3-IAld, with AhR agonistic activity that results in the production of IL-22 and colonization resistance. The 2 pathways thus converge on AhR that, in turn, may potentially regulate IDO1 and ignite a self-sustained AhR-IDO1 activation loop. Collectively, the host and microbial pathways of tryptophan utilization contribute to disease tolerance and homeostasis. Details and abbreviations are described in the text.

Our group has previously identified a microbial pathway of Trp utilization that regulates *Candida* commensalism and mucosal homeostasis in the gut.^33^ Indeed, the commensal *Lactobacilli* (*Lactobacillus reuteri*) could switch to Trp as energy source and produce 3-IAld via the ArAT pathway. Indole-3-aldehyde, in turn, by working as a ligand of AhR, stimulated innate lymphoid cells to release IL-22 that efficiently controlled *Candida albicans* colonization by promoting epithelial integrity and the release of antimicrobial peptides. The scenario emerging from these findings appears to trace the dichotomy of resistance versus tolerance back to the different pathways of Trp utilization (Figure 1). Indeed, the microbial AhR-IL-22 axis appears to promote resistance by means of primitive antifungal defense mechanisms that include the homeostatic maintenance of the triad microbiota, epithelium, and immune system.^62^ On the other hand, the host IDO1 pathway emerges as a functional specialization of antifungal tolerance mechanism that has evolved to facilitate the establishment of a fungal microbiome.^48^ It should also be noted that although the IDO1 product kynurenine and the microbial product 3-IAld are both ligands of AhR, the outcome may be different because AhR activation results in a variety of effects that depend, among others, on the ligand itself.^63^ Therefore, the IDO1 pathway and the microbial Trp metabolism, although intersecting at a common node, may underlie distinct functional activities in the resistance vs tolerance antifungal response.

While promoting different responses, the 2 pathways are not separated, but strictly interconnected. First of all, they share the same substrate, and the equilibrium between pathway utilization might be perturbed. An unbalanced response might lead to disease, not only in the response to a pathogen, but also in a variety of other conditions.^64^ Second, the activity of the 2 pathways is cross-regulated. Indeed, not only kynurenines are ligands of AhR and modulate its activity, as previously stated, but AhR can also regulate IDO1 expression and function. For instance, an autocrine AhR/IL-6/STAT3 signaling loop was shown to sustain IDO1 activity in cancer^65^ and AhR triggers Src-dependent phosphorylation of IDO1 and promotes its signaling activities.^32^ These results would suggest that IDO1 can modulate AhR activity by means of kynurenine production and, in turn, AhR can promote IDO1-dependent functions. It remains to be established whether and how this loop is actually operative and, in this case, the conditions that trigger its emergence. Because various agonists can differently affect AhR activity, it is also unclear which microbial derivatives may actually influence IDO1 expression and function.

Another interesting implication for the AhR-IDO1 cross-regulation relates to IDO2. Indeed, the IDO2 gene contains 2 putative xenobiotic response elements,^66^ thus raising the possibility that AhR might directly regulate the expression of IDO2. However, it is unclear whether IDO2 might contribute to kynurenine production^67^ and, therefore, to the activation of AhR. However, it is possible that IDO2 participates with other mechanisms. For instance, a substantial body of evidence has prompted the concept that IDO2 might act as a proinflammatory mediator in autoimmune diseases and in contact hypersensitivity.^68^ It is unclear whether a similar role is played in fungal responses but, should this be the case, it would open up a scenario in which AhR activation promotes resistance not only by enhancing mucosal barrier function but also by arming a proinflammatory pathway. This possibility warrants further investigation.

## The AhR/IDO1 Pathway: Moving Beyond Infections

As previously stated, a perturbation of Trp utilization by the different metabolic pathways might have implications in several pathological conditions,^64^ including metabolic syndrome (MetSyn) in which an overactivation of the IDO/kynurenine pathway has been observed.^69^ 3-IAld is a metabolite derived from the microbial degradation of the amino acid Trp and represents a “postbiotic” as it is produced by commensal lactobacilli. The interest for postbiotics relates to their favorable safety profile and ability to modulate healthy physiological processes.^70^ We have exploited the therapeutic potential of 3-IAld for the prevention of gut inflammation in experimental MetSyn. MetSyn is characterized by the diagnosis of a cluster of diseases, including visceral obesity, low high-density lipoprotein, hypertension, hyperglycemia and hypercholesterolemia.^71^ MetSyn manifests as a result of leaky intestinal epithelia, also caused by diet-induced dysregulation within the gut microflora. MetSyn is a major risk factor for type 2 diabetes, and treatment is generally in the form of encouragement to adopt healthy lifestyle habits. Unfortunately, such treatment strategies are either ineffective or slow-acting. We have explored the therapeutic utility of 3-IAld loaded into nanoparticles in an experimental model of epithelial barrier dysfunction induced on feeding mice with high-fat diet.^72^ This model recapitulates the MetSyn. We found that 3-IAld was indeed active in the (1) repair of damaged epithelial cells, (2) reduction of inflammation, (3) control of metabolic indexes, and (4) prevention of inflammatory microbial dysbiosis (Borghi et al, manuscript in preparation). As the disruption of the epithelial barrier is implicated in many human diseases, including respiratory allergies, encephalitis, and neurodegenerative diseases, the possibility to target both the host and the microbiota by modulating the cross-regulatory circuit between AhR and IDO1 in human infections and inflammatory diseases makes the microbial indoles, including 3-IAld, one of the greatest challenges in the field.

## Conclusions

The harnessing of the AhR/IDO1 pathway may represent a much-needed strategy for improving and preventing the burden of fungal diseases. By shifting the current view of infection pathogenesis from pathogen- to host-oriented view, we have provided proof-of-concept evidence of the feasibility of therapeutic approaches to reduce infectious disease burden by targeting immunometabolic checkpoints leading to tolerance. In addition, the druggability of this pathway suggests that the exploitation of microbial metabolites to promote homeostasis and microbial symbiosis at mucosal surfaces in infections and inflammatory settings is becoming a reality. Ultimately, the therapeutic use of postbiotics is increasingly being recognized for their excellent safety profile and their ability to rescue gut health while preserving microbiota integrity.^73^ Thus, postbiotics are a teaching example of how mining the microbiota for therapeutic leads may turn to be a great opportunity in modulating cooperative host and microbial defenses.

## References

[1] Medzhitov R Schneider DS Soares MP. Disease tolerance as a defense strategy. Science. 2012;335:936-941. 22363001 10.1126/science.1214935 PMC3564547

[2] Soares MP Teixeira L Moita LF. Disease tolerance and immunity in host protection against infection. Nat Rev Immunol. 2017;17:83-96. 28044057 10.1038/nri.2016.136

[3] Raberg L Graham AL Read AF. Decomposing health: tolerance and resistance to parasites in animals. Philos Trans R Soc Lond B Biol Sci. 2009;364:37-49. 18926971 10.1098/rstb.2008.0184 PMC2666700

[4] Best A White A Boots M. The coevolutionary implications of host tolerance. Evolution. 2014;68:1426-1435. 24475902 10.1111/evo.12368

[5] Smith VH Holt RD. Resource competition and within-host disease dynamics. Trends Ecol Evol. 1996;11:386-389. 21237891 10.1016/0169-5347(96)20067-9

[6] Parada Venegas D De la Fuente MK Landskron G, et al Short chain fatty acids (SCFAs)-mediated gut epithelial and immune regulation and its relevance for inflammatory bowel diseases. Front Immunol. 2019;10:277. 30915065 10.3389/fimmu.2019.00277 PMC6421268

[7] Zeier J. New insights into the regulation of plant immunity by amino acid metabolic pathways. Plant Cell Environ. 2013;36:2085-2103. 23611692 10.1111/pce.12122

[8] Grohmann U Bronte V. Control of immune response by amino acid metabolism. Immunol Rev. 2010;236:243-264. 20636821 10.1111/j.1600-065X.2010.00915.x

[9] Li P Yin YL Li D Kim SW Wu G. Amino acids and immune function. Br J Nutr. 2007;98:237-252. 17403271 10.1017/S000711450769936X

[10] Das P Lahiri A Lahiri A Chakravortty D. Modulation of the arginase pathway in the context of microbial pathogenesis: a metabolic enzyme moonlighting as an immune modulator. PLoS Pathog. 2010;6:e1000899. 20585552 10.1371/journal.ppat.1000899 PMC2887468

[11] Naglik JR Candida immunity. New J Sci. 2014;2014:27.

[12] Ohsugi S Iwasaki Y Takemura Y, et al An inhaled inducible nitric oxide synthase inhibitor reduces damage of Candida-induced acute lung injury. Biomed Res. 2007;28:91-99. 17510494 10.2220/biomedres.28.91

[13] Wagener J MacCallum DM Brown GD Gow NA. Candida albicans chitin increases arginase-1 activity in human macrophages, with an impact on macrophage antimicrobial functions. mBio. 2017;8:e01820-16. 10.1128/mBio.01820-16 PMC5263244 28119468

[14] Philippe B Ibrahim-Granet O Prevost MC, et al Killing of Aspergillus fumigatus by alveolar macrophages is mediated by reactive oxidant intermediates. Infect Immun. 2003;71:3034-3042. 12761080 10.1128/IAI.71.6.3034-3042.2003 PMC155721

[15] Barron L Smith AM El Kasmi KC, et al Role of arginase 1 from myeloid cells in th2-dominated lung inflammation. PLoS ONE. 2013;8:e61961. 23637937 10.1371/journal.pone.0061961 PMC3634833

[16] Lapp K Vodisch M Kroll K, et al Characterization of the Aspergillus fumigatus detoxification systems for reactive nitrogen intermediates and their impact on virulence. Front Microbiol. 2014;5:469. 25309516 10.3389/fmicb.2014.00469 PMC4160965

[17] Badawy AA. Kynurenine pathway of tryptophan metabolism: regulatory and functional aspects. Int J Tryptophan Res. 2017;10:1178646917691938. 28469468 10.1177/1178646917691938 PMC5398323

[18] Roager HM Licht TR. Microbial tryptophan catabolites in health and disease. Nat Commun. 2018;9:3294. 30120222 10.1038/s41467-018-05470-4 PMC6098093

[19] Yano JM Yu K Donaldson GP, et al Indigenous bacteria from the gut microbiota regulate host serotonin biosynthesis. Cell. 2015;161:264-276. 25860609 10.1016/j.cell.2015.02.047 PMC4393509

[20] Kwon YH Wang H Denou E, et al Modulation of gut microbiota composition by serotonin signaling influences intestinal immune response and susceptibility to colitis. Cell Mol Gastroenterol Hepatol. 2019;7:709-728. 30716420 10.1016/j.jcmgh.2019.01.004 PMC6462823

[21] Knecht LD O’Connor G Mittal R, et al Serotonin activates bacterial quorum sensing and enhances the virulence of Pseudomonas aeruginosa in the host. Ebiomedicine. 2016;9:161-169. 27333040 10.1016/j.ebiom.2016.05.037 PMC4972532

[22] Narui K Noguchi N Saito A, et al Anti-infectious activity of tryptophan metabolites in the L-tryptophan-L-kynurenine pathway. Biol Pharm Bull. 2009;32:41-44. 19122278 10.1248/bpb.32.41

[23] Puccetti P Grohmann U. IDO and regulatory T cells: a role for reverse signalling and non-canonical NF-kappaB activation. Nat Rev Immunol. 2007;7:817-823. 17767193 10.1038/nri2163

[24] Munn DH Mellor AL. IDO in the tumor microenvironment: inflammation, counter-regulation, and tolerance. Trends Immunol. 2016;37:193-207. 26839260 10.1016/j.it.2016.01.002 PMC4916957

[25] Munn DH Mellor AL. Indoleamine 2,3 dioxygenase and metabolic control of immune responses. Trends Immunol. 2013;34:137-143. 23103127 10.1016/j.it.2012.10.001 PMC3594632

[26] Fallarino F Grohmann U Puccetti P. Indoleamine 2,3-dioxygenase: from catalyst to signaling function. Eur J Immunol. 2012;42:1932-1937. 22865044 10.1002/eji.201242572

[27] Choera T Zelante T Romani L Keller NP. A multifaceted role of tryptophan metabolism and indoleamine 2,3-dioxygenase activity in Aspergillus fumigatus-host interactions. Front Immunol. 2017;8:1996. 10.3389/fimmu.2017.01996 PMC5786828 29403477

[28] de Araujo EF Feriotti C Galdino NAL Preite NW Calich VLG Loures FV. The IDO-AhR axis controls Th17/Treg immunity in a pulmonary model of fungal infection. Front Immunol. 2017;8:880. 28791025 10.3389/fimmu.2017.00880 PMC5523665

[29] Romani L. Immunity to fungal infections. Nat Rev Immunol. 2011;11:275-288. 21394104 10.1038/nri2939

[30] Stockinger B Di Meglio P Gialitakis M Duarte JH. The aryl hydrocarbon receptor: multitasking in the immune system. Annu Rev Immunol. 2014;32:403-432. 24655296 10.1146/annurev-immunol-032713-120245

[31] Korecka A Dona A Lahiri S, et al Bidirectional communication between the Aryl hydrocarbon Receptor (AhR) and the microbiome tunes host metabolism. NPJ Biofilms Microbiomes. 2016;2:16014. 28721249 10.1038/npjbiofilms.2016.14 PMC5515264

[32] Bessede A Gargaro M Pallotta MT, et al Aryl hydrocarbon receptor control of a disease tolerance defence pathway. Nature. 2014;511:184-190. 24930766 10.1038/nature13323 PMC4098076

[33] Zelante T Iannitti RG Cunha C, et al Tryptophan catabolites from microbiota engage aryl hydrocarbon receptor and balance mucosal reactivity via interleukin-22. Immunity. 2013;39:372-385. 23973224 10.1016/j.immuni.2013.08.003

[34] Bozza S Campo S Arseni B, et al PTX3 binds MD-2 and promotes TRIF-dependent immune protection in aspergillosis. J Immunol. 2014;193:2340-2348. 25049357 10.4049/jimmunol.1400814

[35] Cunha C Aversa F Lacerda JF, et al Genetic PTX3 deficiency and aspergillosis in stem-cell transplantation. N Engl J Med. 2014;370:421-432. 24476432 10.1056/NEJMoa1211161

[36] Moretti S Renga G Oikonomou V, et al A mast cell-ILC2-Th9 pathway promotes lung inflammation in cystic fibrosis. Nat Commun. 2017;8:14017. 28090087 10.1038/ncomms14017 PMC5241810

[37] Renga G Moretti S Oikonomou V, et al IL-9 and mast cells are key players of Candida albicans commensalism and pathogenesis in the gut. Cell Rep. 2018;23:1767-1778. 29742432 10.1016/j.celrep.2018.04.034 PMC5976578

[38] Piliponsky AM Romani L. The contribution of mast cells to bacterial and fungal infection immunity. Immunol Rev. 2018;282:188-197. 29431211 10.1111/imr.12623 PMC5812373

[39] De Luca A Iannitti RG Bozza S, et al CD4(+) T cell vaccination overcomes defective cross-presentation of fungal antigens in a mouse model of chronic granulomatous disease. J Clin Invest. 2012;122:1816-1831. 22523066 10.1172/JCI60862 PMC3336987

[40] Iannitti RG Napolioni V Oikonomou V, et al IL-1 receptor antagonist ameliorates inflammasome-dependent inflammation in murine and human cystic fibrosis. Nat Commun. 2016;7:10791. 26972847 10.1038/ncomms10791 PMC4793079

[41] Borghi M De Luca A Puccetti M, et al Pathogenic NLRP3 inflammasome activity during Candida infection is negatively regulated by IL-22 via activation of NLRC4 and IL-1Ra. Cell Host Microbe. 2015;18:198-209. 26269955 10.1016/j.chom.2015.07.004

[42] de Luca A Smeekens SP Casagrande A, et al IL-1 receptor blockade restores autophagy and reduces inflammation in chronic granulomatous disease in mice and in humans. Proc Natl Acad Sci U S A. 2014;111:3526-3531. 24550444 10.1073/pnas.1322831111 PMC3948220

[43] Moretti S Bozza S Oikonomou V, et al IL-37 inhibits inflammasome activation and disease severity in murine aspergillosis. PLoS Pathog. 2014;10:e1004462. 25375146 10.1371/journal.ppat.1004462 PMC4223056

[44] Jaeger M Carvalho A Cunha C, et al Association of a variable number tandem repeat in the NLRP3 gene in women with susceptibility to RVVC. Eur J Clin Microbiol Infect Dis. 2016;35:797-801. 26951262 10.1007/s10096-016-2600-5 PMC4840230

[45] Oikonomou V Moretti S Renga G, et al Noncanonical fungal autophagy inhibits inflammation in response to IFN-gamma via DAPK1. Cell Host Microbe. 2016;20:744-757. 27889463 10.1016/j.chom.2016.10.012 PMC5161749

[46] Oikonomou V Renga G De Luca A, et al Autophagy and LAP in the fight against fungal infections: regulation and therapeutics. Mediators Inflamm. 2018;2018:6195958. 29692681 10.1155/2018/6195958 PMC5859860

[47] Romani L Oikonomou V Moretti S, et al Thymosin alpha1 represents a potential potent single-molecule-based therapy for cystic fibrosis. Nat Med. 2017;23:590-600. 28394330 10.1038/nm.4305 PMC5420451

[48] Zelante T Fallarino F Bistoni F Puccetti P Romani L. Indoleamine 2,3-dioxygenase in infection: the paradox of an evasive strategy that benefits the host. Microbes Infect. 2009;11:133-141. 19007906 10.1016/j.micinf.2008.10.007

[49] Zhang YJ Reddy MC Ioerger TR, et al Tryptophan biosynthesis protects mycobacteria from CD4 T-cell-mediated killing. Cell. 2013;155:1296-1308. 24315099 10.1016/j.cell.2013.10.045 PMC3902092

[50] Desvignes L Ernst JD. Interferon-gamma-responsive nonhematopoietic cells regulate the immune response to Mycobacterium tuberculosis. Immunity. 2009;31:974-985. 20064452 10.1016/j.immuni.2009.10.007 PMC2807991

[51] Blumenthal A Nagalingam G Huch JH, et al M. tuberculosis induces potent activation of IDO-1, but this is not essential for the immunological control of infection. PLoS ONE. 2012;7:e37314. 22649518 10.1371/journal.pone.0037314 PMC3359358

[52] Schmidt SK Ebel S Keil E, et al Regulation of IDO activity by oxygen supply: inhibitory effects on antimicrobial and immunoregulatory functions. PLoS ONE. 2013;8:e63301. 23675474 10.1371/journal.pone.0063301 PMC3652816

[53] Belkaid Y Hand TW. Role of the microbiota in immunity and inflammation. Cell. 2014;157:121-141. 24679531 10.1016/j.cell.2014.03.011 PMC4056765

[54] Hashimoto T Perlot T Rehman A, et al ACE2 links amino acid malnutrition to microbial ecology and intestinal inflammation. Nature. 2012;487:477-481. 22837003 10.1038/nature11228 PMC7095315

[55] Wikoff WR Anfora AT Liu J, et al Metabolomics analysis reveals large effects of gut microflora on mammalian blood metabolites. Proc Natl Acad Sci U S A. 2009;106:3698-3703. 19234110 10.1073/pnas.0812874106 PMC2656143

[56] Zheng X Xie G Zhao A, et al The footprints of gut microbial-mammalian co-metabolism. J Proteome Res. 2011;10:5512-5522. 21970572 10.1021/pr2007945

[57] Sonowal R Swimm A Sahoo A, et al Indoles from commensal bacteria extend healthspan. Proc Natl Acad Sci U S A. 2017;114:E7506-E7515. 28827345 10.1073/pnas.1706464114 PMC5594673

[58] Lee JH Wood TK Lee J. Roles of indole as an interspecies and interkingdom signaling molecule. Trends Microbiol. 2015;23:707-718. 26439294 10.1016/j.tim.2015.08.001

[59] Esser C Rannug A. The aryl hydrocarbon receptor in barrier organ physiology, immunology, and toxicology. Pharmacol Rev. 2015;67:259-279. 25657351 10.1124/pr.114.009001

[60] Hubbard TD Murray IA Bisson WH, et al Adaptation of the human aryl hydrocarbon receptor to sense microbiota-derived indoles. Sci Rep. 2015;5:12689. 26235394 10.1038/srep12689 PMC4522678

[61] Hubbard TD Murray IA Perdew GH. Indole and tryptophan metabolism: endogenous and dietary routes to ah receptor activation. Drug Metab Dispos. 2015;43:1522-1535. 26041783 10.1124/dmd.115.064246 PMC4576673

[62] Schreiber F Arasteh JM Lawley TD. Pathogen resistance mediated by IL-22 signaling at the epithelial-microbiota interface. J Mol Biol. 2015;427:3676-3682. 26497621 10.1016/j.jmb.2015.10.013

[63] Denison MS Faber SC. And now for something completely different: diversity in ligand-dependent activation of ah receptor responses. Curr Opin Toxicol. 2017;2:124-131. 28845473 10.1016/j.cotox.2017.01.006 PMC5570615

[64] Agus A Planchais J Sokol H. Gut microbiota regulation of tryptophan metabolism in health and disease. Cell Host Microbe. 2018;23:716-724. 29902437 10.1016/j.chom.2018.05.003

[65] Litzenburger UM Opitz CA Sahm F, et al Constitutive IDO expression in human cancer is sustained by an autocrine signaling loop involving IL-6, STAT3 and the AHR. Oncotarget. 2014;5:1038-1051. 24657910 10.18632/oncotarget.1637 PMC4011581

[66] Vogel CF Goth SR Dong B Pessah IN Matsumura F. Aryl hydrocarbon receptor signaling mediates expression of indoleamine 2,3-dioxygenase. Biochem Biophys Res Commun. 2008;375:331-335. 18694728 10.1016/j.bbrc.2008.07.156 PMC2583959

[67] Fatokun AA Hunt NH Ball HJ. Indoleamine 2,3-dioxygenase 2 (IDO2) and the kynurenine pathway: characteristics and potential roles in health and disease. Amino Acids. 2013;45:1319-1329. 24105077 10.1007/s00726-013-1602-1

[68] Merlo LM Mandik-Nayak L. IDO2: a pathogenic mediator of inflammatory autoimmunity. Clin Med Insights Pathol. 2016;9:21-28. 27891058 10.4137/CPath.S39930 PMC5119657

[69] Laurans L Venteclef N Haddad Y, et al Genetic deficiency of indoleamine 2,3-dioxygenase promotes gut microbiota-mediated metabolic health. Nat Med. 2018;24:1113-1120. 29942089 10.1038/s41591-018-0060-4

[70] Wegh CAM Geerlings SY Knol J Roeselers G Belzer C Postbiotics and their potential applications in early life nutrition and beyond. Int J Mol Sci. 2019;20:E4673. 31547172 10.3390/ijms20194673 PMC6801921

[71] Grundy SM Brewer HB Jr Cleeman JI, et al Definition of metabolic syndrome: Report of the National Heart, Lung, and Blood Institute/American Heart Association conference on scientific issues related to definition. Circulation. 2004;109:433-438. 14744958 10.1161/01.CIR.0000111245.75752.C6

[72] Zou J Chassaing B Singh V, et al Fiber-mediated nourishment of gut microbiota protects against diet-induced obesity by restoring IL-22-mediated colonic health. Cell Host Microbe. 2018;23:41-53.e4. 29276170 10.1016/j.chom.2017.11.003 PMC6005180

[73] Paul D Manna S Mandal SM. Antibiotics associated disorders and post-biotics induced rescue in gut health. Curr Pharm Des. 2018;24:821-829. 29283050 10.2174/1381612824666171227221731

